# Evaluation of the In Vivo Biocompatibility of Amorphous Calcium Phosphate-Containing Metals

**DOI:** 10.3390/jfb11020045

**Published:** 2020-06-23

**Authors:** Pio Moerbeck-Filho, Suelen C. Sartoretto, Marcelo J. Uzeda, Maurício Barreto, Alena Medrado, Adriana Alves, Mônica D. Calasans-Maia

**Affiliations:** 1 Implantology Department, Escola Bahiana de Medicina e Saúde Pública, Salvador, Bahia 40290-000, Brazil; piomoerbeck@hotmail.com (P.M.-F.); mauriciobarreto@implo.com.br (M.B.); 2 Oral Surgery Department, Veiga de Almeida University, Rio de Janeiro 20271-020, Brazil; susartoretto@hotmail.com; 3 Oral Surgery Department, Universidade Iguaçu, Nova Iguaçu 26260-045, Brazil; mjuzeda@gmail.com; 4 Oral Surgery Department and Clinical Research Laboratory in Dentistry, Universidade Federal Fluminense, Niteroi 24020-140, Brazil; 5 Oral Pathology Department, Escola Bahiana de Medicina e Saúde Púlbica, Salvador, Bahia 40290-000, Brazil; alenamedrado@hotmail.com; 6 Oral Diagnosis Department, Universidade Federal Fluminense, Niteroi 24020-140, Brazil; aterezinhanovellino@gmail.com

**Keywords:** biocompatibility, calcium phosphate, nanotechnology, metals, mice, ISO standard

## Abstract

Among the biomaterials based on calcium phosphate, hydroxyapatite has been widely used due to its biocompatibility and osteoconduction. The substitution of the phosphate group by the carbonate group associated with the absence of heat treatment and low synthesis temperature leads to the formation of carbonated hydroxyapatite (CHA). The association of CHA with other metals (strontium, zinc, magnesium, iron, and manganese) produces amorphous calcium phosphate-containing metals (ACPMetals), which can optimize their properties and mimic biological apatite. This study aimed to evaluate the biocompatibility and biodegradation of ACPMetals in mice subcutaneous tissue. The materials were physicochemically characterized with Fourier Transform InfraRed (FTIR), X-Ray Diffraction (XRD), and Atomic Absorption Spectrometry (AAS). Balb-C mice (*n* = 45) were randomly divided into three groups: carbonated hydroxyapatite, CHA (*n* = 15), ACPMetals (*n* = 15), and without implantation of material (SHAM, *n* = 15). The groups were subdivided into three experimental periods (1, 3, and 9 weeks). The samples were processed histologically for descriptive and semiquantitative evaluation of the biological effect of biomaterials according to ISO 10993-6:2016. The ACPMetals group was partially biodegradable; however, it presented a severe irritating reaction after 1 and 3 weeks and moderately irritating after nine weeks. Future studies with other concentrations and other metals should be carried out to mimic biological apatite.

## 1. Introduction

Hydroxyapatite (HA) is a naturally occurring mineral, a component of bone and teeth, and a synthesized material with wide application in medicine for the healing of bones. Due to its biocompatibility and bioactivity [1,2,3] HA has been used in dentistry as a bone substitute for alveolar ridge preservation [4], sinus lift elevation [5], and as a drug carrier to prevent and control inflammatory processes, chronic infections, and to enhance the endogenous healing capacity of bone defects, which ultimately result in the significant improvement of bone regeneration [6,7,8,9]. Although, the main limitation of HA ceramics is their high crystallinity, low adsorption capacity for drugs, and poor in vivo bioabsorbability. In a previous study, sintered hydroxyapatite spheres were implanted in rabbit tibia, and after 26, 52, and 78 weeks there was no statistically significant difference between the periods concerning the diameter and area of the spheres, proving their low bioabsorption [10]. This result is due to the thermal treatment at 1100 °C to which this material was subjected, resulting in a highly crystalline structure [1,10]. Therefore, despite its excellent properties, HA has limitations, mainly because it remains in the body for a long time, occupying space in which there should be newly formed bone [11].

To increase the therapeutic capacity of HA, modifications in its chemical composition and the size of the crystals have been studied, making it more similar to natural bone. The ionic substitutions in the phosphate (PO_4_) and hydroxyl (OH) groups, by carbonate (CO_3_), allow the synthesis of carbonated hydroxyapatite (CHA) with new characteristics, subsequently overcoming limitations such as low bioabsorption [3,12,13,14]. Substitution can occur both at the A-site (OH) and the B-site (PO_4_). Habibovic et al. (2010) concluded that type-A carbonated hydroxyapatites showed greater bone neoformation in vivo when compared to type-B [14]. In a study carried out by Landi et al. (2003), porous carbonated hydroxyapatite had twice as much bone as stoichiometric hydroxyapatite, in addition to that, it also had the properties of biocompatibility and bioactivity, and it was more absorbed when compared to HA [3,13].

The number of isomorphic substitutions that hydroxyapatite can undergo incorporating several ions into its structure can optimize its clinical performance, making it more soluble, spatially modifying its crystal structure and favoring its bioabsorption [10] Previous studies have already carried out the partial replacement of calcium by metals such as zinc [10,15,16,17,18,19,20] and strontium [21,22,23,24,25,26] which are capable of optimizing bone repair.

In this context, this study proposes to evaluate the biocompatibility and biodegradation of amorphous calcium phosphate-containing various metals (strontium, zinc, magnesium, iron, and manganese) by means of the subcutaneous implantation of mice, following the standard of evaluation of ISO 10993-6:2016.

## 2. Materials and Methods

### 2.1. Synthesis of Biomaterials

In this study, the samples were composed of particles smaller than 20 nm and ranging from 1.6 < Ca/P < 2.0 of amorphous carbonated hydroxyapatitecalcium phosphate-containing metals (ACPMetals) and samples of carbonated hydroxyapatite (CHA), both synthesized at 37 °C and not sintered.

The first cycle of biomaterial synthesis was carried out through wet precipitation. In essence, calcium nitrate solutions Ca(NO_3_)_2_·4H_2_O (0.151255M) and nitrate of the following metals: Ca(NO_3_)_2_·4H_2_O (0.151255 M), Sr(NO_3_)_2_ (0.009646 M), Zn(NO_3_)_2_·4H_2_O (0.009646 M), (NH_4_)_2_Fe(SO_4_)_2_·6H_2_O (0.001929 M), Mn(NO_3_)_2_·4H_2_O (0.009646 M), Mg(NO_3_)_2_·6H_2_O (0.009646 M) dripped into the reactor through two peristaltic pumps (Masterflex^®^ Pumps, Sigma-Aldrich, São Paulo, Brazil), dibasic ammonium phosphate—(NH_4_)_2_HPO_4_ (0.114599 M) and ammonium carbonate—(NH_4_)_2_CO_3_ (0.001158 M), with partial replacement of the phosphate group by the carbonate group.

The original solutions were adjusted to pH 9.0 by adding ammonium hydroxide solution (NH_4_OH) (99% pure, Merck^®^, Darmstadt, Germany) to an aqueous solution of Ca(NO_3_)_2_ (99% pure, Merck) at 37 °C. The anionic substitution of the group (PO_4_)^3−^ by (CO_3_)^2−^ produces a material with elemental composition very close to the mineral phase of biological apatite [27].

Then, lyophilization was performed for 24 h, obtaining the solid phase of the material, which was macerated and separated by a sieve with a 74 mm mesh opening.

After obtaining the calcium phosphate powders, 100 mL of 1% (*w*/*v*) sodium alginate (Sigma Aldrich Biochemika, Buchs, Switzerland) was added to 15 g of the said powders and mixed until completely homogenization and a ceramic paste was obtained.

### 2.2. Spheres Preparation

The spheres were made using a 24G 1 ¾ needle (BD Precision Glide^®^, Curitiba, PR, Brazil), for the dripping of the biomaterial in the fluid state, in 0.15M calcium chloride solution prepared with Merck reagent. After contact with the solution, an agglutination process began; then, the obtained material was washed with deionized water until the saline solution was eliminated. This process lasted for 24 h.

The spheres obtained were separated in granulometry, through stainless steel sieves with mesh, ranging from 400 to 600 µm, and subsequently, packed in polypropylene flasks (Eppendorf) and sterilized by gamma radiation (cobalt radiator 60). The total dose applied to the samples was 15kGy and the total irradiation time was 760 min. This entire biomaterial synthesis cycle was carried out at the Centro Brasileiro de Pesquisas Físicas (CBPF), Rio de Janeiro, Brazil.

### 2.3. Characterization of Biomaterials

The preparation of samples for the different characterization analyses was conducted according to previous studies [28,29]. 

#### 2.3.1. X-Ray Diffraction (XRD)

X-ray diffraction assessed the crystallinity of the materials and the presence of phases. The analysis used the X-ray diffractor, HZG4 Zeiss, with CuKa radiation (=1.5418 A) with an angle scan of 10–100 and a step of 0.05. The samples were placed on a slide in the form of powder for later reading on the device. The D2 PHASER equipment was used.

#### 2.3.2. Fourier-Transform Infrared Spectroscopy (FTIR)

Infrared vibrational spectroscopy was performed to identify the clusters present in the biomaterials. The infrared spectra for the powder samples were obtained using the Shimadzu Fourier transform spectrophotometer, IR-Prestige 21, with a KBr DTGS detector and a KBr beam separator. Transmittance analysis was performed using tablets with 1% KBr in the median infrared range (4000–400 cm^−1^). The IRTracer-100 FTIR spectrophotometer was used.

#### 2.3.3. Atomic Absorption Spectroscopy (AAS)

This spectroscopy was performed qualitatively and to quantitatively analyze the elements of a sample by means of its electromagnetic or mass spectrum. These transitions present specific wavelengths forming absorption and emission spectra that will identify and quantify the elements present in the sample under analysis. The Shimadzu AA-7000 equipment was used.

### 2.4. In Vivo Analysis

The present study was carried out following the Brazilian guidance for animals use in research and approved by the Ethics Committee on Animal Use (CEUA/UFF: No. 836) of Universidade Federal Fluminense, Niterói, Brazil. Furthermore, this study complied with the Animal Research: Reporting of In Vivo Experiments (ARRIVE) guidelines regarding the relevant items [30,31]. 

### 2.5. Animal Characterization

In this study, 45 Balb/C mice, female and male, weighing 20 to 35 g were used, provided by the Central Animals Laboratory from Universidade Federal Fluminense. The animals were randomly divided according to the experimental groups, CHA, ACPMetals, and without implantation of material (SHAM (clot) (*n* = 15) and experimental periods of 1, 3, and 9 weeks (*n* = 5). During the experimental periods, the animals were kept in mini-isolators and fed with pelleted feed and water at will. Each mini-isolator was lined with dry wood shavings (pine shavings), a completely nontoxic material that was replaced daily to provide animal health and well-being. The animals were kept at a temperature from 20 to 22 °C.

### 2.6. Anesthesia and Surgery Procedures

After fasting for 24 h, all animals were submitted to general anesthesia intraperitoneally with an injection of 0.6 mL of the anesthetic solution prepared with 1.0 mL of 10% Ketamine (Dopalen^®^—100 mg/mL, Ceva Saúde Animal Ltd.a., São Paulo, SP, Brazil), 0.5 mL of 2% xylazine (Anasedan^®^—20 mg/mL, Ceva Saúde Animal Ltd.a.) and 8.5 mL of sterile saline solution (KabiPac^®^, Fresenius Kabi Brasil Ltd.a, São Paulo, SP, Brazil). About three minutes later, trichotomy and disinfection were performed with a disinfectant solution of 2% alcoholic chlorhexidine, followed by the apposition of previously sterilized surgical drapes, for the delimitation and isolation of the surgical site. A rectilinear incision in the dorsal region of each animal was performed with a scalpel cable Nº. 3 (Schwert, A. Schweickhardt GmbH & Co. KG, Tuttlingen, Germany) and blade No. 15C (Surgistar, Joinville, SC, Brazil) about 10 mm in length, followed by muscle fascia skin divulsion with the help of blunt-tip scissors (Schwert, A. Schweickhardt GmbH & Co. KG, Tuttlingen, Germany), exposing the subcutaneous mesh for the insertion of the biomaterial in this region using a standardized meter. This was followed by the suture with nylon thread 5.0 (Ethicon^®^, Johnson & Johnson, Somerville, NJ, USA) and antisepsis with gauze and alcoholic chlorhexidine solution.

Postoperative analgesia was performed with Meloxicam^®^ (EuroFarma, Laboratórios Ltd.a., São Paulo, SP, Brazil) 5 mg/kg subcutaneously every 24 h for three days.

### 2.7. Obtaining the Samples

After the experimental periods of 1, 3, and 9 weeks, the animals of each experimental group received a lethal dose of general anesthetic to collect the samples and surrounding tissues with a 5 mm safety margin.

### 2.8. Sample Processing

The obtained samples were fixed in paraformaldehyde 4% for 48 h, decalcified in Ethylenediamine tetraacetic acid (EDTA) solution (Biodinâmica^®^, Ibiporã, PR, Brazil), dehydrated, clarified, and included in paraffin. Through the blocks obtained, 5 μm thick cuts were made and stained with hematoxylin and eosin (HE) for descriptive and semiquantitative histological evaluation.

### 2.9. Descriptive Histological Analysis

According to ISO 10993-6:2016, each subcutaneous tissue sample was carefully analyzed macro- and micro-scopically to record the extent of each tissue reaction observed. The tissue response was evaluated in the area comprised in the regions surrounding the implantation of the biomaterials and interface.

The slides obtained from the paraffin blocks and stained with HE were observed in a bright-field light microscope (OLYMPUS BX43, Tokyo, Japan). These images were captured by a high-resolution digital camera (OLYMPUS SC100, Tokyo, Japan) using 4, 20, and 40× Acroplan objective lenses.

The descriptive analysis of the tissue response to biomaterials was assessed according to the presence of inflammatory infiltrate, vascular neoformation, amount of biomaterial remaining, and its disposition inside the implanted site.

### 2.10. Analysis of Results According to ISO 10993-6: 2016

From each slide stained with HE, ten photomicrographs were captured (fields run by scanning without overlapping) corresponding to the regions surrounding the implanted biomaterial, using a 40× objective lens (OLYMPUS SC100, Tokyo, Japan) according to ISO 10993-6:2016. The semiquantitative analysis of the tissue response (neovascularization, degree of fibrosis, and fatty infiltrate), and the distribution of inflammatory cells (neutrophils, lymphocytes, plasma cells, macrophages and giant cells), followed the parameters of ISO 10993-6:2016. The difference between the scores of the test and control groups classified the material according to the following criteria: nonirritating (0 to 2.9), mild irritating (3.0 to 8.9), moderately irritating (9.0 to 15, 0) and severe irritant (>15.0) [31]. In Tables S1–S3, the values were totaled according experimental periods, and then an average score for test and control treatments were calculated. 

### 2.11. Statistical Analysis

After a D’Agostino–Pearson normality test, the results of each inflammatory cells response and overall tissue reaction were compared through Student’s *t*-test, considering an alpha error of 5%, using the software GraphPad Prism 7.0 (GraphPad^®^, San Diego, CA, USA).

## 3. Results

### 3.1. X-Ray Diffraction

**Figure 1:**
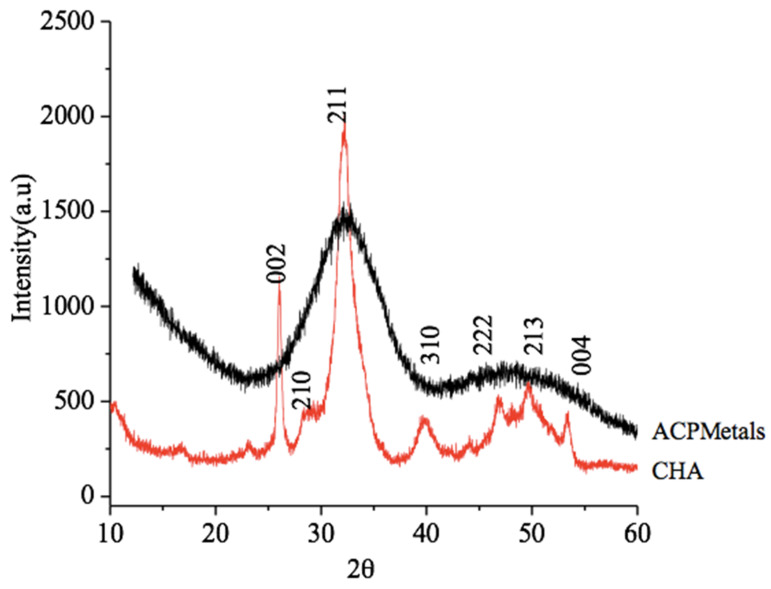
X-ray diffraction (XRD). Diffractogram of the carbonated hydroxyapatite (CHA) and of the amorphous calcium phosphate-containing metals (ACPMetals) groups.

The diffractograms corresponding to the CHA group and ACPMetals are shown in Figure 1. The CHA group showed an X-ray diffraction pattern with peaks that correspond to hydroxyapatite (PCPDFWIN 09.0432 standard). The absence of peaks of ACPMetals group characterizes an amorphous material and with decreased crystallinity compare to CHA. 

### 3.2. Fourier-Transform Infrared Spectroscopy (FTIR)

**Figure 2:**
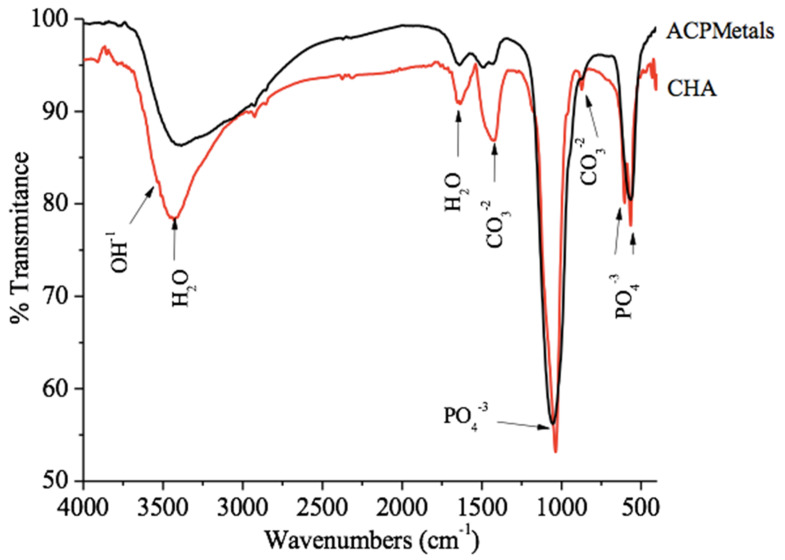
Infrared vibrational spectroscopy with Fourier transform (FTIR). The spectrum of the CHA and of the ACPMetals groups.

The FTIR spectrum of the CHA and ACPMetals groups are constituted by vibrational bands of phosphate groups in 1094 cm^−1^, 1031 cm^−1^, 961 cm^−1^, 607 cm^−1^, 562 cm^−1^. The bands observed in 1444 and 876 cm^−1^ are typical of carbonate ions in the apatite structure, which confirms that the replacement of carbonate ions occurred according to the proposed synthesis. The bands observed at 3574 cm^−1^ and 634 cm^−1^ are characteristic of the hydroxyl group (OH)^−1^ [6] (Figure 2).

### 3.3. Atomic Absorption Spectroscopy

The process of synthesis used in this study yielded an amorphous calcium phosphate-containing metals powder that was composed: 3.52% by wt zinc, 2.46% by wt strontium, 0.78% by wt magnesium, 2.65% by wt manganese, and 4.45% by wt iron. AAS analysis, which is a more accurate test, confirmed that the ACPMetals contained the different metals (Table 1).

### 3.4. Descriptive Histological Analysis

#### 3.4.1. SHAM Group

**Figure 3:**
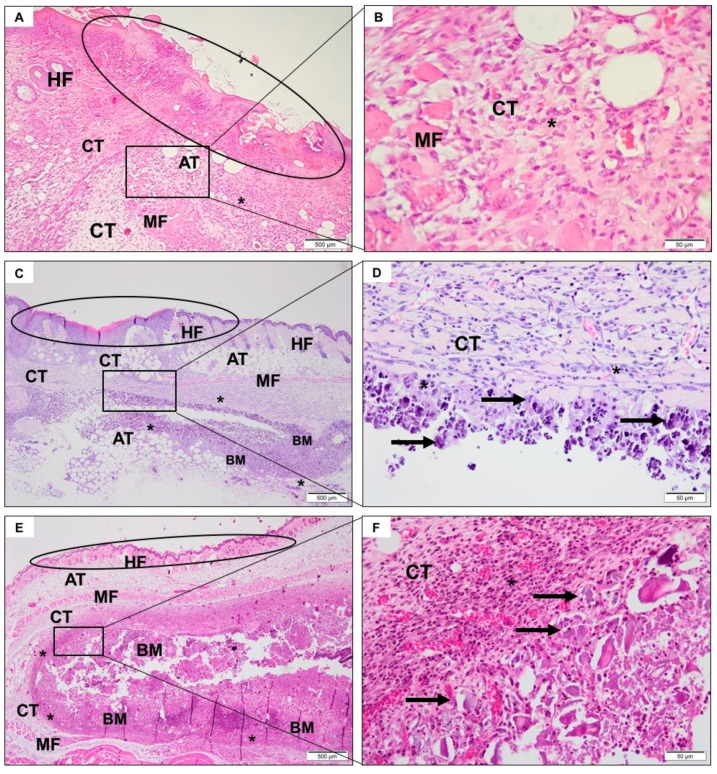
Photomicrographs representative of the region of implantation of the SHAM group (without implantation of material) (**A**,**B**), CHA (**C**,**D**) and ACPMetals (**E**,**F**) group 1 week after implantation. (**A**,**C**,**E**) Circle: epidermis and papillary dermis with hair follicle (HF), recovering connective tissue (CT) with focal intense and inflammatory cells (*); muscle fibers (MF) and adipocyte tissue (AT) are noted. Biomaterial zone (BM), specifically in **C**,**E**. (**B**) Detail in highest objective, disorganized muscular fibers (MF) permeated by connective tissue (CT) and inflammatory infiltrate (*). (**D**,**F**) Detail in highest objective, particulate biomaterial (black arrow) intermediated by connective tissue (CT) with inflammatory mononuclear cells predominantly (*). **A**,**C**,**E**, 40× magnification, scale bar: 500 μm; **B**,**D**,**F**, 400× magnification, scale bar: 50 μm. Hematoxylin and eosin staining.

**Figure 4:**
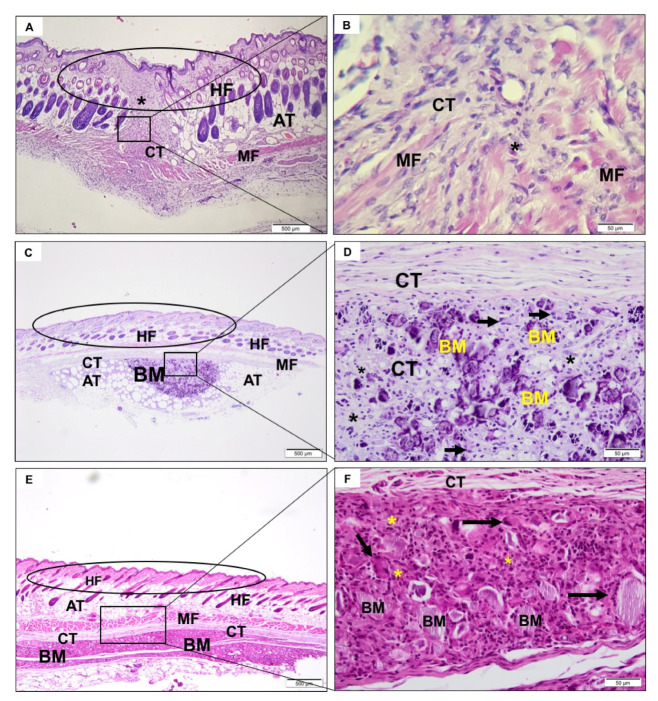
Photomicrographs representative of the region of implantation of the SHAM (**A**,**B**), CHA (**C**,**D**) and ACPMetals (**E**,**F**) group 3 weeks after implantation. (**A**,**C**,**E**) Circle: epidermis and papillary dermis with hair follicle (HF), recovering connective tissue (CT) with inflammatory cells (*); muscle fibers (MF) and adipocyte tissue (AT). Note **C**,**E** granulation reaction in the biomaterial zone (BM). (**B**) Detail in highest objective, collagen and muscle fibers in organization process with moderate inflammatory cells. (**D**,**F**) Detail in highest objective, particulate biomaterial (BM) intermediated by connective tissue (CT) with granulations reaction (*); multinucleated giant cells were observed (black arrow). **A**,**C**,**E**, 40× magnification, scale bar: 500 μm; **B**,**D**,**F**, 400× magnification, scale bar: 50 μm. Hematoxylin and eosin staining.

**Figure 5:**
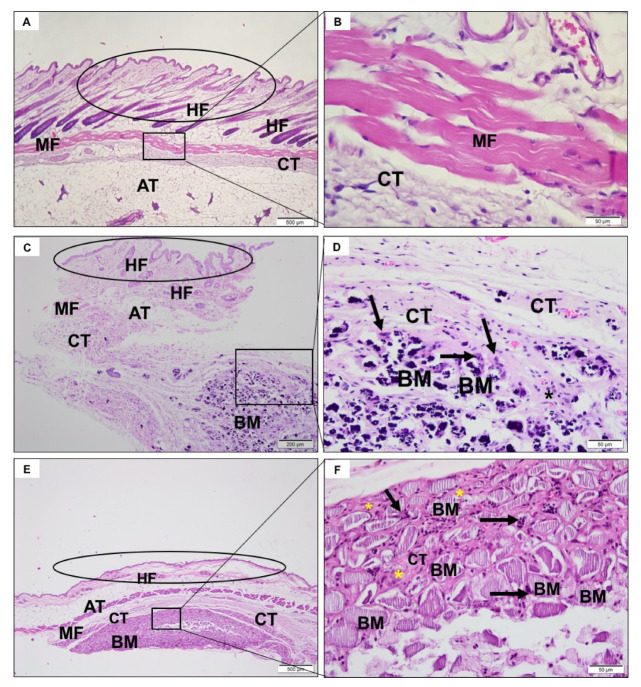
Photomicrographs representative of the region of implantation of the SHAM (**A**,**B**), CHA (**C**,**D**) and ACPMetals (**E**,**F**) group 9 weeks after implantation. (**A**,**C**,**E**) Circle: epidermis and papillary dermis with hair follicle (HF), recovering connective tissue (CT) with inflammatory cells (*); muscle fibers (MF) and adipocyte tissue (AT). Note in **C**,**E** the granulation reaction in the biomaterial zone (BM). (**B**) Detail in highest objective, mature collagen and muscle fibers. **(D**,**F)** Detail in highest objective, particulate biomaterial (BM) intermediated by fibrous connective tissue (CT) inflammatory cells (*) and multinucleated giant cells was observed (black arrow). **A**,**C**,**E**, 40× magnification, scale bar: 500 μm; **B**,**D**,**F**, 400× magnification, scale bar: 50 μm. Hematoxylin and eosin staining.

In the first experimental period, a predominantly neutrophilic inflammatory infiltrate was observed in the incision region, compatible with the surgical procedure (Figure 3A,B). After 3 (Figure 4A,B) and 9 weeks (Figure 5A,B), the tissue was healing with no signs of inflammation.

#### 3.4.2. CHA Group

After a week, it was possible to observe the presence of the biomaterial, without signs of degradation. There was the recruitment of macrophages, multinucleated giant cells, and moderate neutrophilic infiltrate permeating the biomaterial (Figure 3C,D). After three weeks, the spheres were already fragmented and in a smaller volume, presenting giant cells directly involving the biomaterial (Figure 4C,D). In nine weeks, there was no reduction in biomaterial volume compared to the previous period. Furthermore, there was a similar amount of neutrophilic infiltrate and giant cells compared to previous period (Figure 5C,D).

#### 3.4.3. ACPMetals

In the first experimental period, there was the presence of moderate polymorphonuclear inflammatory infiltrates, mononucleated cells, and blood vessels that were distributed concentrically around the implanted biomaterial (Figure 3E,F). The collagen fibers of the connective tissue were organized to “encapsulate” the biomaterial. After 3 weeks, the persistence of granulation tissue, with inflammatory infiltrate, predominantly lymph-plasmacytic, with macrophages and multinucleated giant cells in the middle of blood vessels was observed. This tissue surrounded the biomaterial grafting site. The giant cells seemed to encompass some particles of the biomaterial. The concentric arrangement of collagen fibers persisted (Figure 4E,F). After 9 weeks, morphological characteristics similar to the previous period were observed. There was the persistence of mononuclear inflammatory cells (mainly macrophages and lymphocytes) and multinucleated giant cells adjacent to the biomaterial zone (BM) and organization of thicker collagen bundles that surrounded the grafting site (Figure 5E,F).

### 3.5. Degree of Irritation of Biomaterials According to ISO 10993-6: 2016

**Figure 6:**
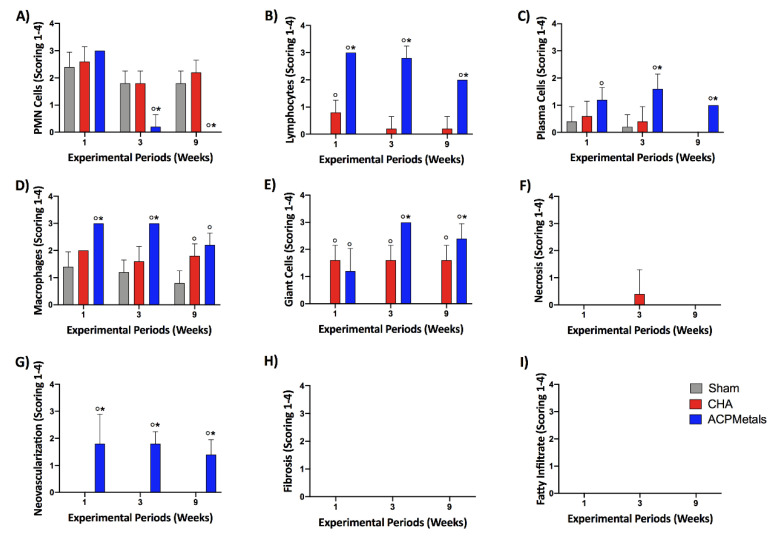
Inflammatory cells response (**A**–**F**) and overall tissue reaction (**G**–**I**). Note that CHA groups responded extremely favorably with only mild increases in inflammation when compared to the negative Sham control. The ACPMetals group presented more intense inflammatory reaction when compared to the other groups at lymphocytes, plasma cells, macrophages, giant cells and neovascularization scorings (data +/− standard deviation; *p* < 0.05); (°) represents significantly greater when compared to the Sham group; (*) represents significantly greater when compared to the CP group.

**Figure 7:**
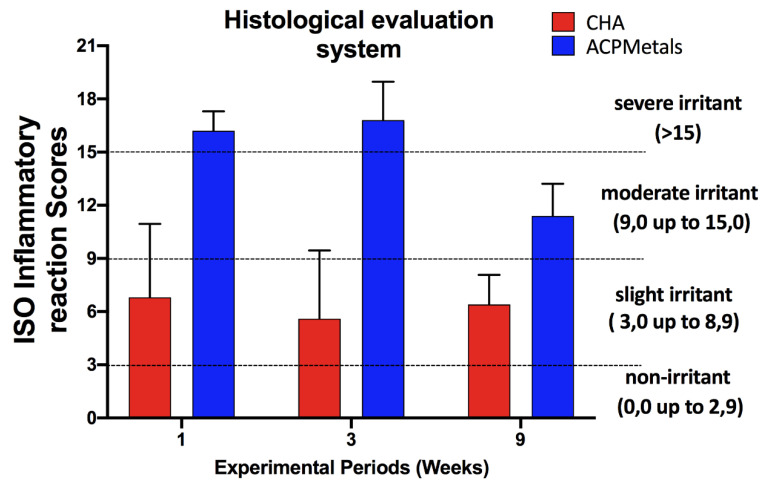
Score of inflammatory reaction according ISO 10993-6:2016. The differences between the scores of the CHA and ACPMetals groups subtracted from the control (SHAM). The groups were ranked according criteria of ISO 10993-6:2016: Nonirritating (0.0 to 2.9); slightly irritating (3.0 to 8.9); moderately irritating (9.0 to 15.0); seriously irritating (>15); the columns represent the mean values.

Following descriptive evaluation of CHA and ACPMetals implanted biomaterials, a semiquantitative analysis was applied using scoring system provided by ISO standard 10993-6. The scores were conducted according to ISO standards to compare the tissue inflammatory response between groups. Figure 6 demonstrates the inflammatory responses of inflammatory cells (Figure 6A–F) as well as overall tissue reaction (Figure 6G–I) with the final calculated outcomes reported in Figure 7. The complete results are presented in Supplemental Tables S1–S3. The SHAM and CHA groups, negative control and positive control, respectively, remained, in the three experimental periods (1, 3 and 9 weeks), in the “mild irritant” classification (from 3 to 8.9 on the ISO scale). The experimental group ACPMetals showed a peak of inflammatory cells in the first and third weeks, when compared to the other two groups, with more significant migration of defense cells to the defect region and is classified as “severe irritant” (>15.0) according to the level provided for by the ISO standard. After nine weeks, the ACPMetals group was considered a “moderate irritant”. 

## 4. Discussion

Animal research, in addition to clinical research, has contributed significantly to finding solutions to biological and biomedical questions and understanding the various physiological and pathological processes affecting humans. To be used as a model, animal species must meet specific criteria of the research’s final goal. This study aimed to evaluate the biocompatibility of a new bone substitute biomaterial, for this, the implantation in the subcutaneous tissue was the first step in this evaluation. Thus, the model of mice was chosen for presenting low cost, secure handling and for allowing the implantation in subcutaneous tissue according to the ISO 10993-6/2016.

There are several methods of preparing HA, such as dry methods, wet methods, high-temperature processes, through biogenic sources, hydrothermal, solid-state reactions, and method combinations. The literature has signaled the prevalence of using the precipitation method; in this study, we prepared the biomaterials by wet precipitation [32,33,34,35]. HA synthesized by the wet method has characteristics similar to HA from dental and bone tissue [36,37]. In a clinical study, where hydroxyapatite was obtained by wet precipitation, histological and radiographic analyses showed better bone growth [38].

During the synthesis, the temperature is an essential factor for the biological response to the biomaterial. It is considered the ideal synthesis temperature between 25 °C to 37 °C to obtain hydroxyapatite with the crystallinity closest to the human bone. Very high temperatures provide low stoichiometry and high crystallinity [39,40].

In this study, we used carbonated hydroxyapatite synthesized at 37 °C, the same temperature used in a previous study [4,5,32]. Patel et al. showed that HA synthesized by lower temperature exhibited a more considerable inflammatory cell and fibroblastic proliferation, with a higher concentration of Ca^2+^ in the ECM, and a difference between them in physical parameters: porosity and crystal morphology [35]. These studies mentioned above [34,39] reinforce the use of low temperatures used in the present study (37 °C).

The biomaterials market points to the use of particles or granules between 400 to 600 µm, where several commercial brands use this range, corroborating with our methodology, where the same range was used [35,41]. In our research, we used the biomaterial in formats of spheres, a form not available on the commercial market. Previous studies showed that sharp edges particles induced a severe inflammatory response in vivo, while no reaction occurred with spherical particles of comparable size. The sphere morphology, allows the maintenance of tissue nutrition and cell population between the spheres, thus avoiding an over-compacted material. Our results corroborate with the studies mentioned above [35,36,37,38,39,40,41,42].

The possibility of incorporating metals into calcium phosphate ceramics for biomedical applications has been discussed in the literature for decades. A significant number of studies on the production of HA and the effect of its ionic substitutions, demonstrate new mechanical, biological, physical, and chemical properties [43,44,45]. Hydroxyapatite can accommodate a large number of elements from the periodic table within its conformity, due to the high flexibility of its structure [44,45]. Specifically, cationic substitutions occur at the sites occupied by Ca^2+^, and the bivalent (i.e., Sr^2+^, Ba^2+^ and Mg^2+^) and also monovalent ones (i.e., Na^+^, K^+^, and others) [45].

The literature points out that strontium (Sr^2+^) is used in treatments for osteoporosis, bone pain, and bone cancer and can exert double effects of bone stimulation. Studies suggest that the incorporation of Sr^2+^ to calcium phosphates seems favorable to bone repair due to its induction properties in bone formation [23,24,46]. In a previous study [26], two concentrations of Sr^2+^ were used, 5 wt% and 10 wt%, and it was found that the lower concentration (5%) had higher resistance to compression; however, the ideal dosage of Sr^2+^ for incorporation to calcium phosphate is still unknown. In our investigation, we used 5 wt% of Sr^2+^; however, it does not corroborate the findings of previous research. 

Researches related to magnesium (Mg^2+^) show their participation in the bone remodeling process [47,48], in bone growth, and osteoblastic activity [49]. A recent study, with magnesium being substituted as a biomaterial, provided 4- to 5-fold improvement to bone repair compared to the control group [49]. Probably due to the association with other metals in our study, different from the previous studies that partially substituted calcium for Sr^2+^ or Mg^2+^, our results do not corroborate with the works described above. 

Some studies have demonstrated the antibacterial potential of zinc (Zn^2+^), inhibiting bacterial and fungal growth, *Escherichia coli*, *Staphylococcus Aureus, Candida Albicans, and Streptococcus Mutans*, however, a content higher than 1000 ppm is needed to exhibit an adequate effect [9,10,11,12,13,14,15,16,17,18,19,20,21,22,23,24,25,26,27,28,29,30,31,32,33,34,35,36,37,38,39,40,41,42,43,44,45,46,47,48,49,50]. 

Consequently, the material with antibacterial properties minimizes the risk of infection and resorption in the remodeling process [20,49]. A recent systematic review found that the concentration of Zn^2+^ can influence 7 to 8% more in bone repair [50]. A previous study of carbonated hydroxyapatite containing Zn^2+^ implanted in the subcutaneous tissue of mice showed that doping with Zn^2+^ did not alter the biocompatibility of the material. However, it did alter bioabsorption [10]. The justification for the use of Zn^2+^ in the present study was based on its bactericidal properties described in previous studies [9,50,51,52].

Iron (Fe^2+^), present in the blood, plays an essential role in cellular metabolic processes and oxygen transport, however, according to the literature, it should be avoided in high concentrations because it affects the disposition of atoms [51,52], corroborating with the present research in which we used 1 wt% of Fe^2+^ concentration.

Mayers et al. (2006), showed that Mn-containing β-tricalcium phosphate (β-TCP) samples were achieved in two ways: a) transformation of precipitated Mn-containing calcium hydroxyapatite (HA) to β-TCP by heating at 1100 °C, and b) preparation by solid-state reaction of a mixture of CaCO_3_, (NH)_2_HPO_4_, and Mn(NO_3_)_2_ at 1100 °C. The authors speculated about the improvement of biocompatibility of HA by (Mn^2+^) in light of the obtained results [53].

Metals as Mn^2+^ and Fe^2+^ have the least scientific evidence associated with HA and were incorporated into the test material to simulate the biological apatite that presents various metals in its constitution and to evaluate how they would behave together.

In the semiquantitative evaluation results of the present study (absent, mild, moderate and severe), although there were no signs of necrosis, it was detected that the inflammatory infiltrates of the experimental group showed a peak that later regressed, which, in a way, was already expected, since the biomaterial is a foreign body with a consequent increase in the migration of defense cells of a short cycle. Based on the ISO standard, this reaction corresponds to the classification “severe irritant” (>15.0), in the periods of 1 and 3 weeks. After 9 weeks in the experimental group, there was a fall in inflammatory cells, and the biomaterial became a “moderate irritant”, according to ISO 10993-6:2016.

On the histological evaluation of ACPMetals, after a week, the presence of a severe polymorphic and mononuclear inflammatory infiltrate and blood vessels that were distributed concentrically around the grafted material, organized to “encapsulate” the biomaterial. After three weeks, the tissue histopathological aspects obtained demonstrated the persistence of granulation tissue, with inflammatory infiltrate, predominantly lymph-plasmacytic, with macrophages and multinucleated giant cells in the middle of blood vessels to encompass the biomaterial.

In the 9th week, there was the persistence of mononuclear inflammatory cells and multinucleated giant cells adjacent to the biomaterial, characterizing this period as a moderate irritant. It is important to emphasize that the presence of multinucleated giant cells and macrophages may be an attempt by the organism to reabsorb the material without necessarily lacking in biocompatibility [49]. From the biological point of view, the presence of these cells around ACPMetals is justified, due to the material being in the form of small spheres and the presence of particles susceptible to phagocytosis. The literature supports that the implantation of synthetic biomaterials based on HA in subcutaneous tissue results in a granulomatous and foreign body reaction at the receptor site [54,55,56].

The association of metals separately has been shown to assist in bone neoformation in several studies [18,19,20,40,50]. However, in the present study, the addition of the five metals together does not show an improvement on the biological response.

According to the results obtained, it is possible to define the biomaterial used as reasonably biodegradable and moderately irritating. The degree of early inflammation was considerable and was importantly considered before evaluation in clinical trials; however, other improvements of biomaterial should be considered before new in vivo studies.

## 5. Conclusions

Throughout the experimental periods evaluated, both groups showed a sphere fragmentation in several small particles. In the ACPMetals group, a severe irritating reaction after 1 and 3 weeks, and moderately irritating after nine weeks was observed. With the results obtained, it was possible to conclude that the association of different ions used in this study did not favor the biological response in the mice’s subcutaneous tissue implantation model. Future studies should be carried out with other metal concentrations to mimic natural apatite.

## Figures and Tables

**Table 1:** Values in moles contained in the structure of the studied biomaterials. Values in moles for calcium (Ca), phosphorus (P), the calcium/phosphorus ratio, and the percentage of metals in the composition of the biomaterial.

Biomaterial	Ca (%)	Mol Ca	P (%)	Mol P	Metals (%)
CP	40.46	1.007	17.72	0.57	-
ACPMetals	28.75	0.72	17.84	0.58	3.52 (Zn) 2.46 (Sr) 0.78 (Mg) 2.65 (Mn) 4.45 (Fe)

## Supplementary Materials

The following are available online at https://www.mdpi.com/2079-4983/11/2/45/s1, Table S1: Semi-quantitative evaluation 1-week post-implantation. All collected data from the semi-quantitative evaluation utilized to evaluate the tissue reaction and biocompatibility as highlighted in ISO 10993-6. The values for each animal are a median of 5 sections evaluated per animal, Table S2: Semi-quantitative evaluation 3 weeks post-implantation. All collected data from the semi-quantitative evaluation utilized to evaluate the tissue reaction and biocompatibility as highlighted in ISO 10993-6. The values for each animal are a median of 5 sections evaluated per animal, Table S3: Semi-quantitative evaluation 9 weeks post-implantation. All collected data from the semi-quantitative evaluation utilized to evaluate the tissue reaction and biocompatibility as highlighted in ISO 10993-6. The values for each animal are a median of 5 sections evaluated per animal.

## References

[1] LeGeros R.Z., Lin S., Rohanizadeh R., Mijares D., LeGeros J.P. (2003). Biphasic calcium phosphate bioceramics: Preparation, properties and applications. J. Mater. Sci. Mater. Electron..

[2] Ono K., Yamamurmo T., Nakamura T., Kokubo T. (1990). Quantitative study on osteoconduction of apatite-wollastonite containing glass ceramic granules, hydroxyapatite granules and alumina ganules. Biomaterials.

[3] Mpc F., Ma B., Mc M., Ds V. (2019). Biological Principles of Nanostructured Hydroxyapatite Associated with Metals: A Literature Review. Insights Biomed..

[4] Resende R.F.B., Sartoretto S.C., Uzeda M.J., Alves A.T.N.N., Calasans-Maia J.A., Rossi A.M., Granjeiro J.M., Calasans-Maia M. (2019). Randomized Controlled Clinical Trial of Nanostructured Carbonated Hydroxyapatite for Alveolar Bone Repair. Materials.

[5] Mourão C.F., Lourenço E.S., Nascimento J.R.B., Machado R.C.M., Rossi A.M., Leite P.E.C., Granjeiro J.M., Alves G.G., Calasans-Maia M.D. (2018). Does the association of blood-derived growth factors to nanostructured carbonated hydroxyapatite contributes to the maxillary sinus floor elevation? A randomized clinical trial. Clin. Oral Investig..

[6] Calasans-Maia M., Junior C.A.B.B., Soriano-Souza C.A., Alves A.T.N.N., Uzeda M.J., Zelaya V.M., Mavropoulos E., Leão M.H.R., De Santana R.B., Granjeiro J.M. (2019). Microspheres of alginate encapsulated minocycline-loaded nanocrystalline carbonated hydroxyapatite: Therapeutic potential and effects on bone regeneration. Int. J. Nanomed..

[7] Parent M., Baradari H., Champion E., Damia C., Viana-Trecant M. (2017). Design of calcium phosphate ceramics for drug delivery applications in bone diseases: A review of the parameters affecting the loading and release of the therapeutic substance. J. Control. Release.

[8] Kolmas J., Krukowski S., Laskus A., Jurkitewicz M. (2016). Synthetic hydroxyapatite in pharmaceutical applications. Ceram. Int..

[9] Martin V., Bettencourt A.F. (2018). Bone regeneration: Biomaterials as local delivery systems with improved osteoinductive properties. Mater. Sci. Eng. C.

[10] Resende R.F., Fernandes G.V.O., Santos S.R.A., Rossi A.M., Lima I., Granjeiro J.M., Calasans-Maia M.D. (2013). Long-term biocompatibility evaluation of 0.5 § zinc containing hydroxyapatite in rabbits. J. Mater. Sci. Mater. Med..

[11] Saijo H., Chung U.-I., Igawa K., Mori Y., Chikazu D., Iino M., Takato T. (2008). Clinical application of artificial bone in the maxillofacial region. J. Artif. Organs.

[12] Landi E., Sprio S., Sandri M., Celotti G., Tampieri A. (2008). Development of Sr and CO3 co-substituted hydroxyapatites for biomedical applications. Acta Biomater..

[13] Landi E., Celotti G., Logroscino G., Tampieri A. (2003). Carbonated hydroxyapatite as bone substitute. J. Eur. Ceram. Soc..

[14] Habibovic P., Juhl M.V., Clyens S., Martinetti R., Dolcini L., Theilgaard N., Van Blitterswijk C. (2010). Comparison of two carbonated apatite ceramics in vivo. Acta Biomater..

[15] Calasans-Maia M., Fernandes G., Rossi A.M., Dias E.P., Almeida G., Mitri F., Granjeiro J.M. (2007). Effect of Hydroxyapatite and Zinc-Containing Hydroxyapatite on Osseous Repair of Critical Size Defect in the Rat Calvaria. Key Eng. Mater..

[16] Fernandes G., Calasans-Maia M., Mitri F., Bernardo V.G., Rossi A.M., Almeida G., Granjeiro J.M. (2008). Histomorphometric Analysis of Bone Repair in Critical Size Defect in Rats Calvaria Treated with Hydroxyapatite and Zinc-Containing Hydroxyapatite 5%. Key Eng. Mater..

[17] Nascimento L., Medeiros M., Calasans-Maia J.A., Alves A., Rossi A.M., Alves G.G., Granjeiro J.M., Calasans-Maia M. (2011). Osseoinduction Evaluation of Hydroxyapatite and Zinc Containing Hydroxyapatite Granules in Rabbits. Key Eng. Mater..

[18] Mayer I., Apfelbaum F., Featherstone J. (1994). Zinc ions in synthetic carbonated hydroxyapatites. Arch. Oral Boil..

[19] Ribeiro S., Sartoretto S.C., Resende R., Uzeda M., Alves A.T., Santos S., Pesce G., Rossi A.M., Granjeiro J.M., Miguel F. (2016). In Vivo Evaluation of Zinc-Containing Nanostructured Carbonated Hydroxyapatite. Key Eng. Mater..

[20] Zhao S.-F., Dong W.-J., Jiang Q.-H., He F.-M., Wang X.-X., Yang G.-L. (2013). Effects of zinc-substituted nano-hydroxyapatite coatings on bone integration with implant surfaces. J. Zhejiang Univ. Sci. B.

[21] Chandran S., Vs H.K., Varma H., John A. (2016). Osteogenic efficacy of strontium hydroxyapatite micro-granules in osteoporotic rat model. J. Biomater. Appl..

[22] Canalis E. (1996). The divalent strontium salt S12911 enhances bone cell replication and bone formation in vitro. Bone.

[23] Valiense H., Barreto M., Resende R.F., Alves A., Rossi A.M., Mavropoulos E., Granjeiro J.M., Calasans-Maia M.D. (2015). In vitro and in vivo evaluation of strontium-containing nanostructured carbonated hydroxyapatite/sodium alginate for sinus lift in rabbits. J. Biomed. Mater. Res. Part B Appl. Biomater..

[24] Carmo A.B.X.D., Sartoretto S.C., Alves A.T.N.N., Granjeiro J.M., Miguel F.B., Calasans-Maia M., Calasans-Maia M.D. (2018). Alveolar bone repair with strontium- containing nanostructured carbonated hydroxyapatite. J. Appl. Oral Sci..

[25] Guo D., Xu K., Zhao X., Han Y. (2005). Development of a strontium-containing hydroxyapatite bone cement. Biomaterials.

[26] Guo D., Hao Y.Z., Fang C.Q., Sun L., Ni P.F., Xu K., Li H.Y., Zhu H., Wang J., Huang X.F. (2013). Influences of Sr dose on the crystal structure parameters and Sr distributions of Sr?incorporated hydroxyapatite. J. Biomed. Mater. Res. Part B Appl. Biomater..

[27] Wopenka B., Pasteris J.D. (2005). A mineralogical perspective on the apatite in bone. Mater. Sci. Eng. C.

[28] Zelaya V.M., Zarranz L., Herrera E.Z., Alves A.T.N.N., Uzeda M.J., Mavropoulos E., Rossi A., Mello A., Granjeiro J.M., Calasans-Maia M. (2019). In vitro and in vivo evaluations of nanocrystalline Zn-doped carbonated hydroxyapatite/alginate microspheres: Zinc and calcium bioavailability and bone regeneration. Int. J. Nanomed..

[29] Sadat-Shojai M., Khorasani M.-T., Dinpanah-Khoshdargi E., Jamshidi A. (2013). Syntesis methods for nanosized hydroxyapatite with diverse structures. Acta Biomater..

[30] Kilkenny C., Browne W., Cuthill I.C., Emerson M., Altman U.G. (2010). Animal research: Reporting in vivo experiments: The ARRIVE guidelines. Br. J. Pharmacol..

[31] Smith A.J., Clutton R.E., Lilley E., Hansen K.E.A., Brattelid T. (2017). PREPARE: Guidelines for planning animal research and testing. Lab. Anim..

[32] Sartoretto S.C., Calasans-Maia M.D., Alves T.N.N., Resende R.F.B., Fernandes C.J.C., Padilha P.M., Rossi A.M., Teti A., Granjeiro J.M., Zambuzzi W.F. (2020). The role of apoptosis associated speck-like protein containing a caspase-1 recruitment domain (ASC) in response to bone substitutes. Mater. Sci. Eng. C.

[33] Michał W., Ewa D., Tomasz C. (2015). Lecithin-based wet chemical precipitation of hydroxyapatite nanoparticles. Colloid Polym. Sci..

[34] Ramirez C., Costa A.M., Bettini J., Ramirez A., Da Silva M.H.P., Rossi A.M., Da Silva M.H.P., Rossi A.M. (2008). Structural Properties of Nanostructured Carbonate Apatites. Key Eng. Mater..

[35] Patel N., Best S.M., Bonfield W., Gibson I.R., Hing K.A., Damien E., Revell P.A. (2002). A comparative study on the in vivo behaviour of hydroxyapatite and silicon substituted hydroxyapatite granules. J. Mater. Sci. Mater. Med..

[36] Ślósarczyk A., Paszkiewicz Z., Paluszkiewicz C. (2005). FTIR and XRD evaluation of carbonated hydroxyapatite powders synthesized by wet methods. J. Mol. Struct..

[37] Narasaraju T.S.B., Phebe D.E. (1996). Some physico-chemical aspects of hydroxylapatite. J. Mater. Sci..

[38] Gomes D.S., Santos A.M.C., Neves G.D.A., Menezes R.R. (2019). A brief review on hydroxyapatite production and use in biomedicine. Cerâmica.

[39] Hesaraki S., Nazarian H., Pourbaghi-Masouleh M., Borhan S. (2013). Comparative study of mesenchymal stem cells osteogenic differentiation on low-temperature biomineralized nanocrystalline carbonated hydroxyapatite and sintered hydroxyapatite. J. Biomed. Mater. Res. Part B Appl. Biomater..

[40] Guo L., Huang M., Zhang X. (2003). Effects of sintering temperature on structure of hydroxyapatite studied with Rietveld method. J. Mater. Sci. Mater. Electron..

[41] O’Donnell C.J., Rao D.S.P., Battese G.E. (2007). Metafrontier frameworks for the study of firm-level efficiencies and technology ratios. Empir. Econ..

[42] Lebre F., Sridharan R., Sawkins M.J., Kelly D.J., O’Brien F.J., Lavelle E.C. (2017). The shape and size of hydroxyapatite particles dictate inflammatory responses following implantation. Sci. Rep..

[43] Grégoire M., Orly I., Menanteau J. (1990). The influence of calcium phosphate biomaterials on human bone cell activities. Anin vitroapproach. J. Biomed. Mater. Res..

[44] Rigo E.C.S., Gehrke S.A., Carbonari M. (2007). Síntese e caracterização de hidroxiapatita obtida pelo método de precipitação. Rev. Dent. Press Periodontia Implantol..

[45] Santos M.L., Florentino A.O., Saeki M.J., Aparecida A.H., Fook M.V.L., Guastaldi A.C. (2005). Síntese de hidroxiapatita pelo método sol-gel utilizando precursores alternativos: Nitrato de cálcio e ácido fosfórico. Eclética Química J..

[46] Hao Y., Yan H., Wang X., Zhu B., Ning C., Ge S. (2012). Evaluation of osteoinduction and proliferation on nano-Sr-HAP: A novel orthopedic biomaterial for bone tissue regeneration. J. Nanosci. Nanotechnol..

[47] Costa N.M., Yassuda D.H., Sader M.S., Fernandes G.V.D.O., Soares G.D., Granjeiro J.M. (2016). Osteogenic effect of tricalcium phosphate substituted by magnesium associated with Genderm® membrane in rat calvarial defect model. Mater. Sci. Eng. C.

[48] Bigi A., Foresti E., Gregorini R., Ripamonti A., Roveri N., Shah J.S. (1992). The role of magnesium on the structure of biological apatites. Calcif. Tissue Int..

[49] Cai Y.L., Zhang J.J., Zhang S., Venkatraman S., Zeng X.T., Du H.J., Mondal D. (2010). Osteoblastic cell response on fluoridated hydroxyapatite coatings: The effect of magnesium incorporation. Biomed. Mater..

[50] Cruz R., Calasans-Maia J.A., Sartoretto S., Moraschini V., Rossi A.M., Louro R.S., Granjeiro J.M., Calasans-Maia M.D. (2018). Does the incorporation of zinc into calcium phosphate improve bone repair? A systematic review. Ceram. Int..

[51] Zuo K.-H., Zeng Y.-P., Jiang D. (2012). Synthesis and magnetic property of iron ions-doped hydroxyapatite. J. Nanosci. Nanotechnol..

[52] Pon-On W., Meejoo S., Tang M. (2007). Incorporation of iron into nano hydroxyapatite particles synthesized by the microwave proces. Int. J. Nanosci..

[53] Mayer I., Cuisinier F.J.G., Popov I., Schleich Y., Gdalya S., Burghaus O., Reinen D. (2006). Phase Relations Between β-Tricalcium Phosphate and Hydroxyapatite with Manganese(II): Structural and Spectroscopic Properties. Eur. J. Inorg. Chem..

[54] Zambuzzi W., Oliveira R.C., Pereira F.L., Cestari T.M., Taga R., Granjeiro J.M. (2006). Rat subcutaneous tissue response to macrogranular porous anorganic bovine bone graft. Braz. Dent. J..

[55] Lee S., Choi H., Shim J.-S., Chung M.-K., Park Y.-B. (2015). Comparative study of recombinant human bone morphogenetic protein-2 carriers in rat subcutaneous tissues: Pilot study. Tissue Eng. Regen. Med..

[56] Avula M.N., Rao A.N., Mcgill L.D., Grainger D.W., Solzbacher F. (2014). Foreign body response to subcutaneous biomaterial implants in a mast cel deficient Kit murine model. Acta Biomater..

