# Supplementary material for: Evaluation of the In Vivo Biocompatibility of Amorphous Calcium Phosphate-Containing Metals
Source: J Funct Biomater. 2020 Jun 23;11(2):45. doi: 10.3390/jfb11020045 (PMC7353583; doi:10.3390/jfb11020045)
Supplement: Supplementary file 1 [file jfb-11-00045-s001.pdf]

Supplementary File

Evaluation of the in Vivo Biocompatibility of Amorphous Calcium Phosphate-Containing Metals

Table S1. Semi-quantitative evaluation 1-week post-implantation. All collected data from the semi-quantitative evaluation utilized to evaluate the tissue reaction and biocompatibility as highlighted in ISO 10993-6. The values for each animal are a median of 5 sections evaluated per animal.

1 week	SHAM					CHA					ACPMetals				
Animals	1	2	3	4	5	1	2	3	4	5	1	2	3	4	5
Polymorphonuclear	2	3	2	2	3	2	3	3	3	2	3	3	3	3	3
Lymphocytes	0	0	0	0	0	0	1	1	1	1	3	3	3	3	3
Plasma Cells	1	0	0	0	1	0	1	0	1	1	2	1	1	1	1
Macrophages	2	1	1	2	1	2	2	2	2	2	3	3	3	3	3
Giant Cells	0	0	0	0	0	1	2	2	1	2	0	2	1	1	2
Necrosis	0	0	0	0	0	0	0	0	0	0	0	0	0	0	0
Score from inflammatory cells SUBTOTAL (X 2)	10	8	6	8	10	10	18	16	16	16	22	24	22	22	24
Neovascularization	0	0	0	0	0	0	0	0	0	0	3	1	1	1	3
Fibrosis	0	0	0	0	0	0	0	0	0	0	0	0	0	0	0
Fatty infiltrate	0	0	0	0	0	0	0	0	0	0	0	0	0	0	0
Sub-total from tissue response	0	0	0	0	0	0	0	0	0	0	3	1	1	1	3
TOTAL	10	8	6	8	10	10	18	16	16	16	25	25	23	23	27
AVERAGE	8,4					15,2					24,6				
RESULT (Test - Control)	---					6,8					9,4				

Table S2. Semi-quantitative evaluation 3 weeks post-implantation. All collected data from the semi-quantitative evaluation utilized to evaluate the tissue reaction and biocompatibility as highlighted in ISO 10993-6. The values for each animal are a median of 5 sections evaluated per animal.

3 weeks	SHAM					CHA					ACPMetals				
Animals	1	2	3	4	5	1	2	3	4	5	1	2	3	4	5
Polymorphonuclear	2	1	2	2	2	2	2	1	2	2	1	0	0	0	0
Lymphocytes	0	0	0	0	0	0	0	1	0	0	3	2	3	3	3
Plasma Cells	0	0	0	0	1	0	1	0	0	1	2	1	1	2	2
Macrophages	1	1	1	1	2	2	2	2	1	1	3	3	3	3	3
Giant Cells	0	0	0	0	0	2	2	1	2	1	3	3	3	3	3
Necrosis	0	0	0	0	0	0	0	0	2	0	0	0	0	0	0
Score from inflammatory cells SUBTOTAL (X 2)	6	4	6	6	10	12	14	10	14	10	24	18	20	22	22
Neovascularization	0	0	0	0	0	0	0	0	0	0	2	2	1	2	2
Fibrosis	0	0	0	0	0	0	0	0	0	0	0	0	0	0	0
Fatty infiltrate	0	0	0	0	0	0	0	0	0	0	0	0	0	0	0
Sub-total from tissue response	6	4	6	6	10	0	0	0	0	0	2	2	1	2	3
TOTAL	6	4	6	6	10	12	14	10	14	10	26	20	21	24	25
AVERAGE	6,4					12					23,2				
RESULT (Test - Control)	---					5,6					11,2				

Table S3. Semi-quantitative evaluation 9 weeks post-implantation. All collected data from the semi-quantitative evaluation utilized to evaluate the tissue reaction and biocompatibility as highlighted in ISO 10993-6. The values for each animal are a median of 5 sections evaluated per animal.

9 weeks	SHAM					CHA					ACPMetals				
Animals	1	2	3	4	5	1	2	3	4	5	1	2	3	4	5
Polymorphonuclear	2	2	2	1	2	2	2	2	2	3	0	0	0	0	0
Lymphocytes	0	0	0	0	0	0	0	1	0	0	2	2	2	2	2
Plasma Cells	0	0	0	0	0	0	0	0	0	0	1	1	1	1	1
Macrophages	1	1	0	1	1	2	2	2	1	2	2	2	2	2	3
Giant Cells	0	0	0	0	0	1	2	1	2	2	2	3	2	3	2
Necrosis	0	0	0	0	0	0	0	0	0	0	0	0	0	0	0
Score from inflammatory cells SUBTOTAL (X 2)	6	6	4	4	6	10	12	12	10	14	14	16	14	16	16
Neovascularization	0	0	0	0	0	0	0	0	0	0	1	1	2	2	1
Fibrosis	0	0	0	0	0	0	0	0	0	0	0	0	0	0	0
Fatty infiltrate	0	0	0	0	0	0	0	0	0	0	0	0	0	0	0
Sub-total from tissue response	0	0	0	0	0	0	0	0	0	0	1	1	2	2	1
TOTAL	6	6	4	4	6	10	12	12	10	14	15	17	16	18	17
AVERAGE	5,2					11,6					16,6				
RESULT (Test - Control)	---					6,4					5				
